# Antioxidant Strategy to Prevent Simulated Microgravity-Induced Effects on Bone Osteoblasts

**DOI:** 10.3390/ijms21103638

**Published:** 2020-05-21

**Authors:** Caterina Morabito, Simone Guarnieri, Alessandra Cucina, Mariano Bizzarri, Maria A. Mariggiò

**Affiliations:** 1 Department of Neuroscience, Imaging and clinical Sciences—Center for Advanced Studies and Technology (CAST), University G. d’Annunzio of Chieti-Pescara, 06100 Chieti, Italy; cmorabit@unich.it (C.M.); simone.guarnieri@unich.it (S.G.); 2 Department of Surgery “Pietro Valdoni”, Sapienza University of Rome, 00161 Rome, Italy; alessandra.cucina@uniroma1.it; 3 Azienda Policlinico Umberto I, 00161 Rome, Italy; 4 Department of Experimental Medicine, Sapienza University of Rome, Systems Biology Group Lab, 00161 Rome, Italy; mariano.bizzarri@uniroma1.it

**Keywords:** osteoblasts, microgravity, random positioning machine, oxidative stress, intracellular calcium

## Abstract

The effects induced by microgravity on human body functions have been widely described, in particular those on skeletal muscle and bone tissues. This study aims to implement information on the possible countermeasures necessary to neutralize the oxidative imbalance induced by microgravity on osteoblastic cells. Using the model of murine MC3T3-E1 osteoblast cells, cellular morphology, proliferation, and metabolism were investigated during exposure to simulated microgravity on a random positioning machine in the absence or presence of an antioxidant—the 6-hydroxy-2,5,7,8-tetramethylchroman-2-carboxylic acid (Trolox). Our results confirm that simulated microgravity-induced morphological and metabolic alterations characterized by increased levels of reactive oxygen species and a slowdown of the proliferative rate. Interestingly, the use of Trolox inhibited the simulated microgravity-induced effects. Indeed, the antioxidant-neutralizing oxidants preserved cell cytoskeletal architecture and restored cell proliferation rate and metabolism. The use of appropriate antioxidant countermeasures could prevent the modifications and damage induced by microgravity on osteoblastic cells and consequently on bone homeostasis.

## 1. Introduction

Bone physiology is featured by a complex crosstalk between different cell phenotypes and their interaction with organic matrix and inorganic matter. In this scenario, biophysical interactions and mechanical loading have a key role in regulating bone tissue metabolism and functional balance of bone remodeling [1]. On Earth, body weight by 1 g-gravity and muscle activities are the main forces that drive bone functional adaptation. This is impaired in microgravity conditions where body weight and muscle activities are deeply modified [2,3]. Considering the increasing interest in space colonization and the number of human missions to explore the deep-space environment, the importance of preserving bone health is becoming a crucial task. The main macroscopic effect induced by microgravity exposure on bone tissue is the decrease of bone mass. In particular, bone mass loss is more evident in bones of legs and the lower back, possibly due both to the unloading and body fluid shifting, and consequently, the decreased blood perfusion [4,5,6].

These effects are due to microgravity-induced modifications at cellular levels that in bone tissue may result in an impairment in the activities and signaling of osteoclasts, osteoblasts, and osteocytes. In particular, numerous studies observed in real and in simulated microgravity (s-microgravity) conditions an increase of bone resorption by osteoclasts and a decrease of bone formation by osteoblasts [7]. These latter ones are among the main actors during bone remodeling and repair [8]. Many experiments were performed in space and on ground in s-microgravity conditions, using different osteoblast-like cell cultures. The obtained results showed that microgravity induced inhibitory effects on cell proliferation and differentiation, and alterations of gene expression profiles [9,10,11,12].

The most commonly used osteoblast-like cell line, as an in vitro model to study the processes of bone formation, remodeling, and healing in physiological and pathological conditions, are the murine MC3T3-E1 cells [13]. For this reason, they become a useful model also to monitor signals triggered by weightlessness. It has been reported that microgravity induced significant modifications on MC3T3-E1-cell growth, morphology, and cell functions. In particular, during spaceflight, in these cells, the cell number, glucose utilization, and prostaglandin E2(PGE2) synthesis were reduced [14]. When MC3T3-E1 were exposed to s-microgravity conditions, using the random positioning machine (RPM), the expression levels of various markers of osteogenesis and the formation of mineralized nodules were suppressed [15,16].

Many Authors focalized their attention on cell cytoskeleton organization, because the cytoskeleton has been reported to be an initial gravity sensor, a mechano-sensitive structure that transduces the variations of external mechanical forces into intracellular chemical signals [17]. On the other hand, the cytoskeleton is also one of the cellular targets of the gravity variations. Luo and colleagues reported that after 12 and 24 h of exposure on RPM, MC3T3-E1 cell viability decreased, cytoskeleton microfilaments were disrupted and concentrated on the cell periphery [18]. The reorganization and the architecture of the cytoskeleton and the shape of MC3T3-E1 osteoblasts changed likewise under microgravity conditions simulated by 2D clinostats [19] and diamagnetic levitations [20].

Another common intracellular target of microgravity is the metabolic pathway. Microgravity affects the oxidative status of bone, increasing the production of reactive oxygen species (ROS) and consequently inducing oxidative stress [21,22].

However, increased levels of ROS had opposite effects on osteoblast and osteoclast cell physiology. ROS induce the apoptosis of osteoblasts and osteocytes and favor osteoclastogenesis. Antioxidants’ treatments counteract the action of oxidants and contribute to activate the differentiation of osteoblasts, the mineralization process and the reduction of osteoclast activity [23].

In a tail-suspension model (a microgravity in vivo model), Morikawa and colleagues reported that mechanical unloading significantly increased intracellular ROS production exacerbating bone loss as a result of reduced osteoblastic abilities. In the same model, the administration of vitamin C, a known antioxidant molecule, significantly attenuated bone loss during unloading [24]. Comparing in vitro and in vivo experiments, some Authors revealed a protective effect of antioxidants. Xin and colleagues reported that the presence of curcumin inhibited unloading-induced ROS formation, enhancing osteoblastic differentiation, and attenuating osteoclastogenesis in hind-limb suspension experiments [25]. The same authors showed counteracting effects of curcumin on the oxidative stress induced by s-microgravity in MC3T3-E1 and RAW cells cultured in the rotating wall vessel bioreactor [25]. Sun and colleagues showed that the treatment with molecular hydrogen alleviated microgravity-induced ROS production preventing bone loss in a hindlimb-suspension rat model, preserving osteoblastic differentiation in MC3T3-E1 cells and reducing osteoclast differentiation in RAW264.7 cells [26].

Considering this evidence, it is clear that microgravity, by altering redox balance, breaks the equilibrium between bone formation and bone absorption.

Nowadays it is essential to prevent bone loss by adopting efficient countermeasures against microgravity-induced injuries.

Herewith, by using the model of murine MC3T3-E1 osteoblast cells exposed to RPM-generated s-microgravity, we focused our attention on 6-hydroxy-2,5,7,8-tetramethylchroman-2-carboxylic acid (Trolox), a well-known water-soluble analogue of vitamin E acting as a scavenger of free radicals. Trolox can neutralize the harmful effects of oxidative stress thus counteracting morphological changes and intracellular triggered signals (Ca^2+^ and ROS levels) evoked by s-microgravity exposure.

## 2. Results

### 2.1. Cell Morphology and Proliferation under s-Microgravity Conditions

The MC3T3-E1 cells were cultured up to 96 h under s-microgravity (exposed cells, RPM) or at 1 g (control cells, Ctr) conditions. At selected times (24, 48, 72, and 96 h) morphological parameters, cell viability, and expression levels of main cytoskeleton proteins were assayed.

**Figure 1:**
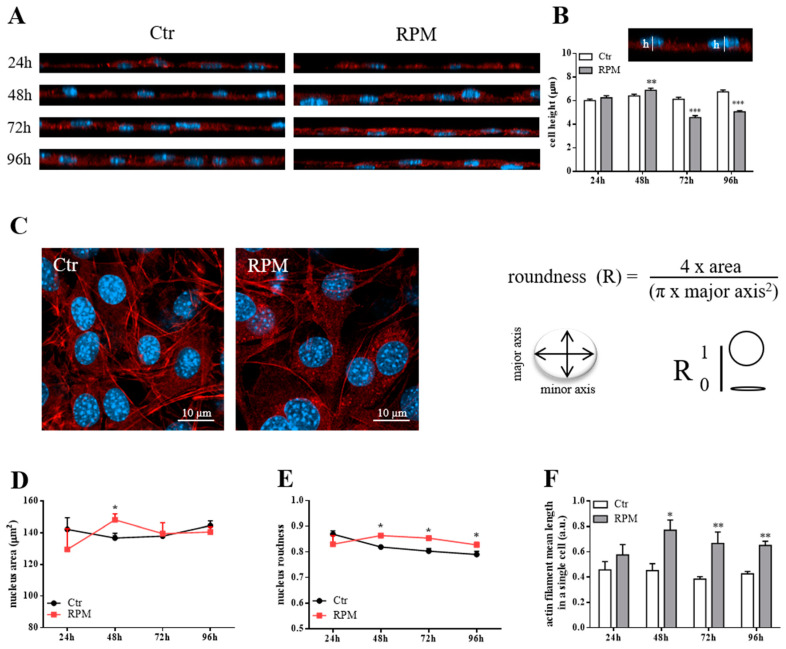
Morphological assessment of MC3T3-E1 cells exposed to s-microgravity conditions. (**A**) Representative orthogonal projections (XZ) obtained from the z-stack images from control cells at 1g (Ctr) or cells exposed to s-microgravity (RPM) at different times (24, 48, 72, and 96 h), stained with Alexa Fluor 546 Phalloidin (for f-actin) and DAPI (for nuclei). (**B**) The graph shows quantitative analyses of cell height. This was calculated as described in Materials and Methods and as shown in the example image (h = height). (**C**) Representative single optical section-images of Ctr or RPM cells, stained with Alexa Fluor 546 Phalloidin (for f-actin) and DAPI (for nuclei), and the formula used to assay nuclei roundness. (**D**–**F**) Quantitative analyses of nucleus area, nucleus roundness, and the mean length of actin filaments of Ctr or RPM cells at different exposure times (24–96 h). The data in the graphs are presented as the means ± SEM from three independent experiments. * *p* < 0.05 vs. *Ctr*; ** *p* < 0.01 vs. *Ctr*, *** *p* < 0.001 vs. *Ctr*.

Figure 1 shows representative images (XZ projection- or single optical section-images) of control and s-microgravity-exposed cells (see also z-stack reconstruction-images in Supplementary Figure S1).

**Figure 2:**
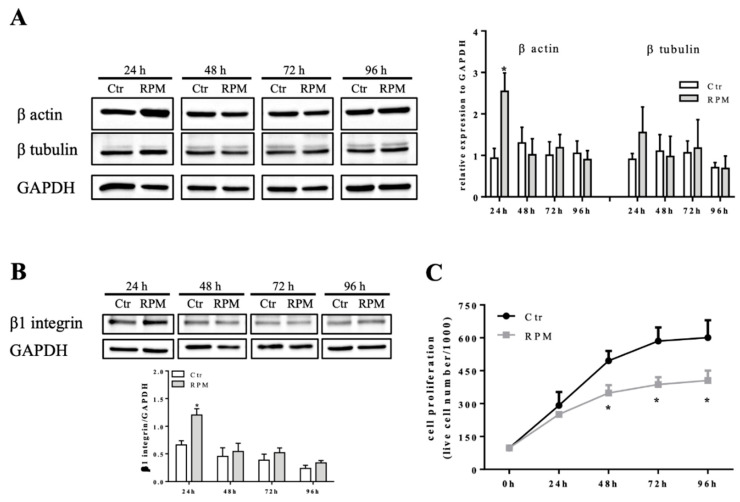
Modulation of cell mechanosensors and proliferation. (**A**,**B**) Representative immunoblots of β actin, β tubulin, and β1 integrin expression levels in extracts from control cells at 1g (Ctr) or cells exposed to s-microgravity (RPM) at different exposure times (24–96 h). The densitometric analyses are plotted as the relative expression calculated as a ratio between the optical density (OD) × mm^2^ of each band and OD × mm^2^ of the corresponding GAPDH band, used as loading control. (**C**) Cell proliferation tested on Ctr and RPM-exposed cells at different exposure times (24–96 h). The data are presented as the means ± SEM from three independent experiments. * *p* < 0.05 vs. *Ctr*.

**Figure 3:**
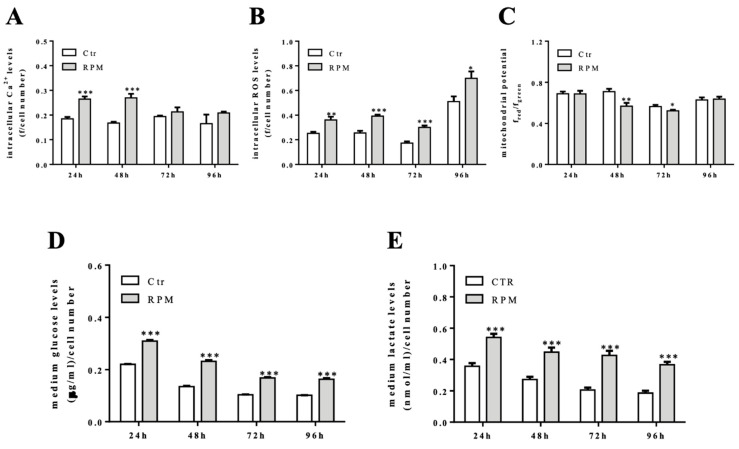
Intracellular and metabolic responses of MC3TE-E1 cells to s-microgravity. (**A**,**B**) Intracellular Ca^2+^ and reactive oxygen species (ROS) levels in live cells grown at 1 g (Ctr) or exposed to s-microgravity (RPM) at different exposure times (24–96 h) and expressed as a ratio between fluorescence emission (**f**) and the number of loaded cells at each time point. (**C**) Mitochondrial membrane potential values graphed as a ratio between red and green fluorescence (**f**) at each time point. (**D**,**E**) Glucose and lactate levels measured in the growth medium of the Ctr and RPM-exposed cells. The data in the graphs are presented as the means ± SEM from three independent experiments. * p < *0.05*, ** p < *0.01*, *** p < *0.001vs.* Ctr.

**Figure 4:**
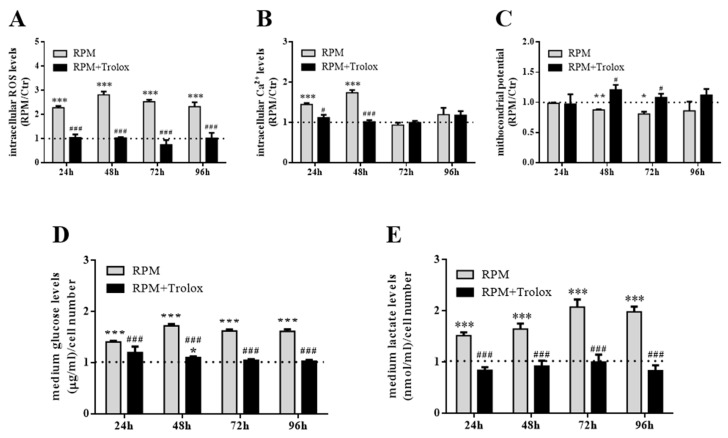
Antioxidant supplementation as countermeasure to s-microgravity. (**A**–**C**) Intracellular ROS, Ca^2+^ levels and mitochondrial membrane potential in live cells exposed to s-microgravity up to 96 h without (RPM) or with Trolox (RPM + Trolox). (**D**,**E**) Glucose and lactate levels measured in growth medium of cells exposed to RPM, in absence of RPM, or presence of 100 µM Trolox (RPM + Trolox). The data in the graphs are presented as the ratio between each parameter and the respective control (dashed line) at each time point, and are the means ± SEM from three independent experiments. * p < *0.05,* ** p < *0.01,* *** p < *0.001vs.* respective Controls; # *p < *0.05,** ### *p < *0.001vs.* RPM+DMSO*. See the complete data sets and detailed statistical analyses, reported in Supplementary Tables (Tables S1–S5).

The quantitative analyses revealed that the 48h-exposure time was a key time for morphological changes. In comparison to control cells, there were the following findings: (1) a small but significant increase of cell height in 48h-exposed cells followed by a decreased height after 72 and 96h-exposure (Figure 1B); (2) a bigger nuclear area in 48h-exposed cells (Figure 1C,D); (3) a significant increase in roundness values of nuclei starting from 48h-exposure (Figure 1C,E); (4) an increased mean length of actin filaments starting from 48h-exposure (Figure 1F). The effects observed at 48h-exposure were probably dependent on the higher expression levels of β actin and β tubulin (even if this latter one did not reach the statistical significance), main components of the cytoskeleton, in 24h-exposed cells in comparison to control cells (Figure 2A). Furthermore, in 24h-exposed cells, an increased expression levels of β1 integrin, a cell adhesion molecule responsible of cell-matrix contacts, was observed (Figure 2B).

Starting from 48h-exposure to s-microgravity, MC3T3-E1 cells significantly decreased the proliferative rate in comparison to control cells (Figure 2C). The following 72h- and 96h-exposures did not affect the proliferative trend (Figure 2C), in addition, the trypan blue-exclusion test revealed a very low percentage of blue-stained cells at any time both in controls and s-microgravity-exposed cells (<5%).

### 2.2. Intracellular Cell Responses to s-Microgravity

The 24h- and 48h-exposure to s-microgravity were also critical for biochemical cell behaviour. Starting from 24h-exposure, the MC3T3-E1 cells cultured on RPM showed increased intracellular Ca^2+^ and ROS levels, in comparison to control cells (Figure 3A,B). Intracellular Ca^2+^ levels regained control values at 72h- and 96h-exposure, while ROS levels were found to be increased up to 96h-exposure (Figure 3B). The cells appeared to react to this metabolic status because mitochondrial membrane potential decreased at 48h- or 72h-exposure, showing signs of mitochondria suffering (Figure 2D). The inference of s-microgravity conditions on the metabolic status of MC3T3-E1 cells was also evident considering the increased levels of glucose and lactate in the culture media during all exposure times (from 24 up to 96 h) (Figure 3D,E).

### 2.3. Counter-Measures to s-Microgravity

**Figure 5:**
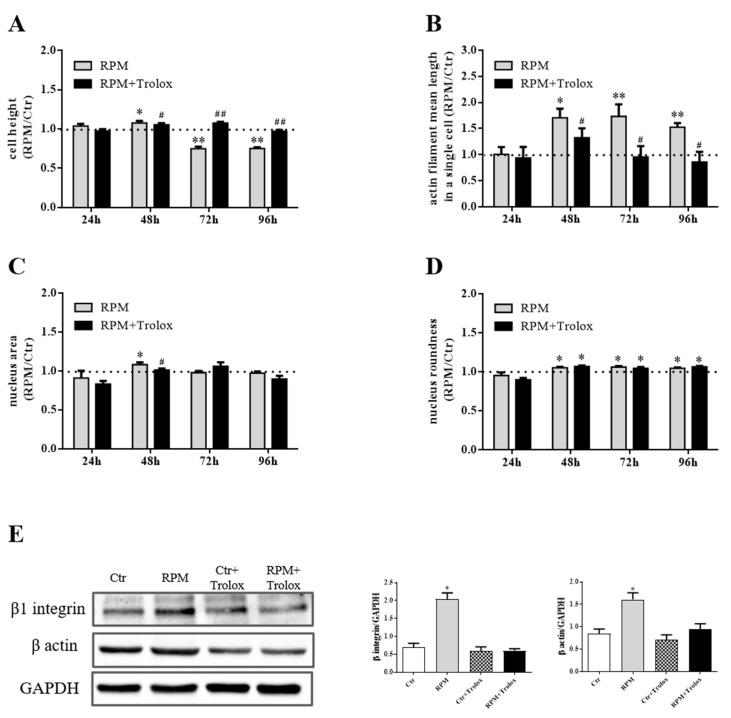
Trolox’s effects as countermeasures to biological changes induced by s-microgravity. (**A**,**B**) Cell height and mean length of actin filaments of cells exposed to s-microgravity up to 96 h of cell culture with (RPM + Trolox) or without (RPM) 100 µM Trolox. (**C**,**D**). Quantitative analyses of nuclei area and roundness of the RPM and RPM + Trolox cells up to 96 h of cell culture. The data A–D are presented as the ratio between each parameter and the respective control (dashed line) at each time point. (**E**) Representative immunoblots β1 integrin and β actin expression levels and the corresponding densitometric analyses of the 1g controls cells without (Ctr) or with 100 µM Trolox (Ctr + Trolox), and s-microgravity-exposed cells without (RPM) or with (RPM + Trolox) 100 µM Trolox up to 24 h of cell culture. The densitometric analyses are plotted as the relative expression calculated as the ratio between the optical density (OD) × mm^2^ of each band and OD × mm^2^ of the corresponding anti-glyceraldehyde-3-phosphatedehydrogenase (GAPDH) band, used as loading control. In all graphs, the data are presented as the means ± SEM from three independent experiments. * p < *0.05,* ** p < *0.01vs.* respective Controls; # *p < *0.05,**## *p < *0.01vs.* RPM+DMSO*. See the complete data sets and detailed statistical analyses, reported in Supplementary Tables (Tables S6–S10 and Western blot analysis).

**Figure 6:**
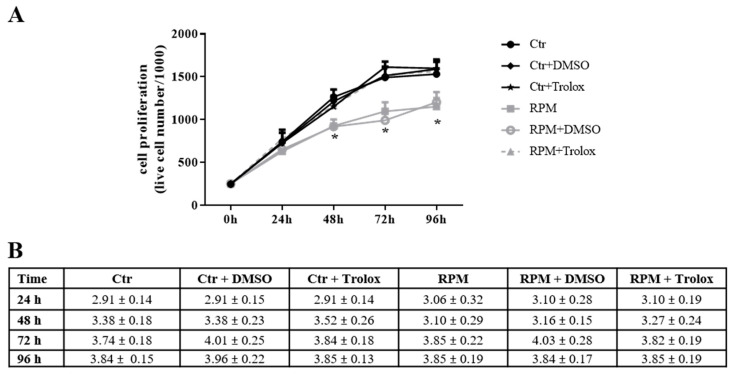
Cell proliferation restoring by Trolox. (**A**) Proliferation of live cells cultured, up to 96 h, at 1 g (Ctr) and s-microgravity (RPM) in absence or presence (+ Trolox) of 100 µM Trolox or its vehicle solution (1 µL in 1 mL of medium) (+ DMSO). (**B**) Thypan Blue-stained cells, considered dead cells, in the same cell populations tested in A. Data are expressed as cell percentage and are presented as the means ± SEM from three independent experiments. * p < *0.05vs.* respective Controls. Complete data sets and statistical analyses are reported in Supplementary Tables (Table S11).

The effects on cell metabolism described above suggested that s-microgravity induced an altered oxidative status in exposed cells. To prevent oxidative unbalance, MC3T3-E1 cells were exposed to s-microgravity in the presence of Trolox to test if this antioxidant could neutralize the morphological and protein expression changes showed in Figure 1 and Figure 2. The data showed in Figure 4, Figure 5 and Figure 6 point out that the presence of the antioxidant counteracted the s-microgravity-induced effects. In particular, starting from 24h-incubation, in RPM-exposed cells, Trolox restored intracellular ROS and Ca^2+^ levels, the mitochondrial membrane potential, as well as glucose and lactate levels in the medium (Figure 4A–E). This evidence was supported by the complete data sets and statistical analyses reported in Supplementary Tables.

We observed that under ROS-evoked stress, not only both cytoskeleton architecture and cell shape are altered, but a wide range of essential biological cell processes, including cell adhesion, proliferation, and survival, are influenced [27,28]. We suggest that increase in ROS levels can likely trigger these changes. Indeed, the presence of Trolox in the medium of cells cultured on RPM almost completely abolished the effects induced by s-microgravity on cell height, the mean length of actin and the expression levels of β actin and β1 integrin (Figure 5A–C,E). However, antioxidant addition did not succeed in completely counteracting the s-microgravity-induced effects on the nucleus shape, as nucleus roundness was unaffected by treatment (Figure 5D).

The antioxidant activity of Trolox was also able to restore the proliferation rate when the molecule was present in the medium of cells exposed to s-microgravity. In addition, the trypan blue-exclusion test revealed a very low percentage (<5%) of blue-stained cells at any times in all tested cell populations (controls and s-microgravity-exposed cells also in the presence of Trolox). These percentages were not significantly different in controls and s-microgravity-exposed cells. The trypan blue-exclusion test is a non-specific assay and can reveal early-necrotic or late-apoptotic cells and, even if we did not perform further investigations regarding this aspect, possible apoptotic or autophagic mechanisms could be involved.

## 3. Discussion

Gravity, as well as many biophysical forces and constraints, plays a critical role in shaping bone structure and functions [29,30,31]. Namely, weightlessness promotes an imbalance between bone formation and resorption that can lead to bone loss [32,33,34]. The development of countermeasures capable of preventing this condition may ensure safe space missions for astronauts, while shedding light on a number of bone pathological ailments, akin to osteoporosis.

The osteoblasts, together with osteoclasts, are critically important for bone formation and remodeling in response to mechanical stimuli [35]. Numerous studies showed that weightlessness affects osteoblast morphology, proliferation, differentiation, gene expression, and oxidative status [22,30].

In the present study, we used the murine MC3T3-E1 cells as model, a widely accepted in vitro osteoblast-like phenotype, and the RPM device to simulate microgravity conditions. This choice was due to previously published reports that consider RPM as a useful, handy, and valid device able to simulate microgravity and to expose adherent cells [36,37]. The experiments were performed on proliferating MC3T3-E1 cells in the absence of any growth factor or differentiating stimulus in order to assess a mere relationship between cellular changes and variation of external forces.

In our experimental condition, the short-term exposure (24–48 h) of MC3T3-E1 cells at s-microgravity is critical for cell behavior, and the presence of an antioxidant preserves the cells. Our data revealed that s-microgravity conditions dramatically modify the cell and nucleus morphology of MC3T3-E1 osteoblasts. Early response includes cell increased height followed, after 72 h, by a more flattened shape, while nuclei adopt a more circular form as their roundness increased. It is well accepted that shape parameters are instrumental in allowing specific cell functions and cell fate commitment towards differentiation [38,39,40].

Several studies, carried out by adopting different weightlessness devices as well as on board the International Space Station, provide similar results. MC3T3-E1 cells exposed to weightlessness in high magnetic gradient environment changed into a flattened shape, their nucleus was enlarged and the cells appeared polygonal-shaped compared to those under 1 g condition [20]. The fractal analyses on MC3T3-E1 cells exposed to RPM revealed that these cells became larger and acquired a more rounded phenotype [12]. Also during parabolic flight shape changes were noticed even if microgravity occurred for very short-lasting time exposure (20 s) [41].

There are also growing evidence that the cell-microenvironment cross-talk can transduce mechanic stimuli through the integrin-cytoskeleton network, which, in turn, transmits physical stresses to nucleoskeleton, eventually leading to remodeling of chromatin structure and nuclear morphology [42,43,44].

In our model, after 24h-exposure to s-microgravity, there were transient increases in integrin and actin expression levels, whereas actin-filament mean length increased after 48h-exposure. Interestingly, this cytoskeleton reorganization is accompanied by the inhibition of cell proliferation, another macroscopic event that became significant only after 48-exposure time.

Testa and colleagues showed that the s-microgravity on RPM induced in murine osteoblasts a significant increase in the mean surface area and roundness, accompanied by a slow-down of cell proliferation, a concomitant increase in cell apoptosis and a reduction of expression level of β1-integrin [12]. In addition, studies conducted in MC3T3-E1 cells exposed to s-microgravity using 2/3D clinostats, showed cytoskeleton alterations accompanied by loss of stress fibers, decreased β-actin mRNA levels [19]. Some apparently contradictory data related to the time-course of the effects of microgravity on changes of cell shape and the different organization and expression of cytoskeleton-integrin networking could depend on several conditions: real or simulated microgravity environment, times of observation, and the different cellular models used (culture systems with specific supports, media, cell density etc).

In another cell model represented by TCam-2—an adherent seminoma cell line—after 24h-exposure to s-microgravity, we observed a modulation of cell shape, and cytoskeletal architecture associated with changes in cell metabolic status [45]. We hypothesized that also in adherent MC3T3-E1 cells there might be the same metabolic response. Indeed, after 24h-exposure to weightlessness, these cells showed reduced extracellular glucose uptake and increased lactate production, indicating an increase of anaerobic metabolic rate. This metabolic shift is accompanied by an increase in Ca^2+^ and ROS levels. It is reported that increased ROS levels inhibit osteoblast functions suppressing osteoblast differentiation via phospholipase C-γ1/extracellular signal-regulated kinase 1/2/NF-KB signaling [46], and promote apoptosis in osteoblasts, that resulted protected by estrogenic treatment [47], therefore influencing the pathophysiology of bone loss [48]. It is well known that bone health status is the result of a balance among different bone cell phenotypes and their interaction with organic matrix and inorganic matter [1]. Consequently, the physiological dynamics of cell architecture and metabolic homeostasis are required to prevent bone alterations. In particular the former is important for cell compartmentalization, division as well as for cell-cell and cell-matrix interactions. The latter represents not only cell energy supply, but also the cell response to extracellular stimuli [35]. It has been noticed that microgravity increases the production of ROS and foster oxidative stress [22,45,49]. Conversely, some reports demonstrated the osteogenic benefits induced by the administration of several antioxidants acting against oxidative stress [50,51]. Based on this evidence, countermeasures have been adopted to restore physiological intracellular redox status [22]. In our experimental conditions, we used as antioxidant Trolox, a water-soluble analogue of vitamin E. The presence of Trolox in RPM-exposed cells counteracted the s-microgravity-induced increase of intracellular ROS levels, restoring both cell metabolic and proliferative rate, while promoting a normalization of their morphological main descriptors. Considering these results, we hypothesize that the alterations observed in RPM-exposed MC3T3-E1 osteoblasts may be the result of the increased ROS levels. ROS act as messengers in the transduction of certain cues (e.g., metabolic and environmental) that influence different signaling and functional pathways [52]. Calcium signaling can be regulated also by ROS production [53], and changes in Ca^2+^ trafficking may affect, in turn, a number of cellular processes, including cell morphology, proliferation, and metabolism. From our results, we can speculate that the ROS increase, induced by s-microgravity, could affect the function (or even the expression) of membrane glucose transporters resulting in a decrease of glucose uptake. This, in turn, could alter cellular metabolic pathways leading to an increase of lactate production and a decrease of mitochondria functioning proven by the reduction of mitochondria membrane potential. Further experiments are need to elucidate this aspect and to detail the intracellular pathways triggered by the s-microgravity exposure. Our data open the way to identify those intracellular reactions that produce an increase of ROS levels in response to s-microgravity. In this scenario. some enzymes could be involved such as the NADPH oxidases present in different cell structures and compartments. Furthermore, considering the effect of s-microgravity on cell proliferation, also possible mechanisms related to this aspect should be explored such as cyclins’ turnover, apoptosis, and autophagocytosis, all processes affected by the cell oxidative status. Other interesting aspects to be evaluated could be to monitor metabolic changes during osteogenesis in bone cells (also from human origin) during exposure to s-microgravity.

In our model, Trolox inhibited the increase in both ROS and Ca^2+^, ultimately “protecting” osteoblasts from the disruptive microgravity-related effects. Overall, these findings suggest that changes in ROS and Ca^2+^ likely play a pivotal role in transducing weightlessness effects into cell physiology. Thereby, the use of appropriate antioxidant countermeasures could prevent the modifications and damage induced by microgravity on osteoblastic cells and so on bone homeostasis. Studies are warranted to deepen knowledge regarding the intracellular sources of increased ROS delivery in weightlessness to find pertinent protective strategies in support of astronaut’s health.

## 4. Materials and Methods

### 4.1. Equipment and Cell Exposure Parameters

The s-microgravity was established using a desktop RPM connected to a control console through standard electrical cables (Dutch Space, Leiden, The Netherlands). The main characteristics of this apparatus were previously described [45,54,55] and the use parameters reported in the manufacturer’s manual. Briefly, the RPM is a 3D clinostat whose running exerts continuous changes in orientation of the biological sample in order to minimize the gravitational force vector. The desktop RPM used in this study, works within a CO_2_ incubator at 37 °C and 95% humidity.

### 4.2. Reagents and Materials

Unless indicated otherwise, reagents and materials were purchased as follows: cell culture medium, sera, and antibiotics from ThermoFisher Scientific (Monza, Italy), cell culture plastic ware from Becton Dickinson Falcon (Steroglass S.r.l., San Martino in Campo, Italy), and the chemicals and standards from Merck Life Science S.r.l. (Milan, Italy).

### 4.3. Cell Cultures

The murine MC3T3-E1 line (ATCC, CRL-2596TM, American type culture collection ATCC-LGC Standards, Teddington, UK), was cultured in growth medium (alpha minimal essential medium, a-MEM) supplemented with 10% foetal bovine serum, 200 mM L-glutamine, 100 IU/mL penicillin and 100 µg/mL streptomycin. After twenty-four hours from cell seeding (10 × 10^3^ cells/cm^2^), all experiments were performed on cells growing up to 96 h at 1 g or at s-microgravity on the RPM in the same incubator and culture conditions. MC3T3-E1 cells were plated as follows: in special-optic 96-well plates (Corning-Costar, Milan, Italy) for measurements of mitochondrial membrane potential and levels of intracellular Ca^2+^ or ROS; in dishes (ø 3.5 cm) or 25 cm^2^ flasks for trypan blue exclusion tests, Western blots analyses, measurements of glucose and lactate levels; on glass coverslips positioned in 3.5 cm-dishes for immunofluorescence staining. Where indicated, cells were grown at 1 g or on RPM in the presence of 100 µM Trolox. This agent was solubilized in dimethyl sulphoxide at concentration of 100 mM than diluted in cell medium at a final concentration of 100 µM. At the beginning of each experimental set, pilot experiments were performed using vehicle administration (1 µL dimethyl sulphoxide in 1 mL medium) to exclude its possible effect or toxicity on cells. All cell culture holders (microplates, flasks, etc.) were completely filled with culture medium to avoid air bubbles and to minimize liquid flow, and consequently buoyancy and shear stress during rotation.

### 4.4. Trypan Blue Exclusion Test

The trypan blue exclusion assay tested cell viability. A trypan blue dye solution (0.5% in phosphate-buffered saline, PBS) stained the cells that were counted using a Bürker chamber. The non-viable MC3T3-E1 cells appeared completely blue-stained.

### 4.5. Fluorescence Staining

The MC3T3-E1 cells were processed at room temperature as follows: fixed with a 4% paraformaldehyde solution in PBS (15 min), washed in PBS, permeabilized with a 0.1% TritonX-100 solution (10 min), stained with Alexa Fluor 546 Phalloidin (1:100 dilution, cod. A22283, ThermoFisher Scientific, Monza, Italy) (1 h); the nuclei were counterstained with DAPI (10 µM, cod. D1306, ThermoFisher Scientific) (10 min). For each sample, images were acquired in 5 different randomly selected fields from 3 independent experiments, using a Zeiss LSM800 URGB confocal system equipped with an upright Axio Observer Z1 microscope, a 40X N.A. 1.3 oil immersion objective and the ZEN Blue 2.1 software (Carl Zeiss, Jena, D). Z scan acquisitions were performed at resolution of 512 × 512 pixel acquiring the focal planes every 0.420 µm using as starting-point the glass adherent side of the cells, and as end-point the plane where the cells vanished from the acquisition field. The actin filament lengths were analyzed using ImageJ software (image processing program, National Institute of Health, Bethesda, MD, USA).

### 4.6. Quantitative Analysis of Immunofluorescence Staining

#### 4.6.1. Cell Height

Cellular height was calculated off-line using the ZEN Blue 2.1 software (Carl Zeiss Spa) from orthogonal projections (XZ) obtained from the Z-stack images acquisition. Measurements of each cell in the acquired field derived tracing the distance between the region of cell adhesion to the glass slide and the highest point of the same cell in proximity of the nuclear region. The quantitative analysis was carried out on at least 5 randomly selected fields from each experimental condition performed in triplicate.

#### 4.6.2. Nuclear Morphometric Analysis

Quantitative analyses (nuclear area and roundness) were performed using FIJI distribution of ImageJ (ver1.52p) [56]. Nuclear area was calculated as the total number of pixels presented in a single nucleus and expressed in square micrometers (1 pixel  =  0.156 µm^2^). Nuclear roundness was calculated using the following formula: 4 × area/(π × major axis^2^), in which the major axis was one of the best fitted ellipse to a given object. A high value for roundness indicates a round nucleus, instead a low value indicates a rather elongated nucleus [57]. The quantitative analysis was carried out on at least 5 randomly selected fields from each experimental condition performed in triplicate.

### 4.7. Actin Filament Length

This parameter was calculated using FIJI distribution of ImageJ (ver1.52p), optical sections deriving from equatorial plane were firstly processed to obtain a simplified morphological model binarizing and skeletonizing using the “make binary” and “skeletonize” functions respectively. On the obtained images the function “Analyze Skeleton (2D/3D)” calculated the averaged actin filament length in a cell expressed as an arbitrary unit (a.u.). The quantitative analysis of the actin filament length was carried out on at least 5 randomly selected fields from each experimental condition performed in triplicate.

### 4.8. Western Blotting

The MC3T3-E1 cells were collected and, after sonication in a cold buffered solution (50 mM Tris-HCl, pH 7.4, 100 mM NaCl, 50 mM NaF, 40 mM -glycerophosphate, 5 mM EDTA, 1% Triton X-100, 200 µM sodium orthovanadate,100 µg/mL PMSF, 10 µg/mL leupeptin, 5 µg/mL pepstatin A, and 10 µg/mL benzamidine), centrifuged (10,000× *g*, 10 min at 4 °C). The supernatants were recovered and the protein concentrations were determined using a protein assay kit (Bio-Rad DC; Bio-Rad, Segrate, Italy).

Cell extracts (40 µg of protein/lane) were loaded on 8% or 10% (*w*/*v*) homogeneous SDS-PAGE slab gels and then transferred to nitrocellulose membranes (Protran; Whatman-GE Healthcare, Milan, Italy). Membranes were hybridized with primary antibodies: a rabbit polyclonal anti-β1 Integrin (1:500 dilution, clone M106 cod. sc-8978, Santa Cruz Biotechnology, Heidelberg, Germany), mouse monoclonal anti-β tubulin (1:500 dilution, clone TBN06 (Tub 2.5) cod. MA5-11732, ThermoFisher Scientific), or anti-β actin antibody (1:1000 dilution, clone 2A3 cod. sc-517582, Santa Cruz Biotechnology), followed by incubation with horseradish-peroxidase-conjugated anti-mouse or anti-rabbit IgGs (1:10,000 dilution, cod. NA931 or cod. NA934 respectively, GE Healthcare, Cologno Monzese, Italy). The relevant proteins were detected using chemiluminescence kits (GE Healthcare), and the signals were acquired and analyzed using an image acquisition system (Uvitec mod Alliance 9.7, Uvitec, Cambridge, UK). A mouse monoclonal anti-glyceraldehyde-3-phosphatedehydrogenase (GAPDH) antibody (1:10,000 dilution, cod. AB2302, Merck S.p.a) was used to reveal GAPDH expression as loading control. The choice of GAPDH as internal standard was due to the experimental evidence (from our and other laboratories [58]) showing that the expression levels of this protein did not change in any condition we tested (1 g or s-microgravity exposure at different times).

### 4.9. Measurements of Glucose and Lactate Levels

The measurements of glucose and lactate levels in the growth media were assayed according to the manufacturer instructions, using a Free Style Optium glucometer (Abbot Laboratories, Rome, Italy) and a LactatePro Analyzer (Arkray Inc. Kyoto, Japan), respectively.

### 4.10. Spectrofluorometric Measurements

The MC3T3-E1 cells, after 40-min incubation at 37 °C, in NES (Normal External Solution: 140 mM NaCl, 2.8 mM KCl, 2 mM CaCl_2_, 2 mM MgCl_2_, 10 mM glucose and 10 mM HEPES, pH 7.3) containing one of the probes (ThermoFisher Scientific) reported in Table 1, were rinsed and fluorescence intensity was detected using a microplate reader (Synergy H1 multimode, Biotek, Bad Friedrichshall, Germany) [45,54].

The fluorescence values (from Fluo-4- or H_2_-DCFDA- loaded cells) were expressed as the means (±SEM) of fluorescence values recorded during 5-min acquisition. Fluorescence values of JC1-loaded cells were expressed as the means (±SEM) of the red/green fluorescence ratio, which depends on the probe chemical status in relation to the mitochondrial membrane potential. For each sample, three independent experiments were performed, each containing eight repetitions.

### 4.11. Statistical Analyses

The statistical analyses were reported in the legend of each figure. Experimental data are expressed as means ± SEM. Statistical significance was calculated by Student’s t-test (Prism5 software, GraphPad, San Diego, CA, USA). *p* values < 0.05 were considered statistically significant.

## Figures and Tables

**Table 1:** Fluorescence probes used for intracellular analyses.

Probe	Excitation (nm)	Emission (nm)	Analyses
Fluo4–AM 5 µM	488	520	Intracellular Ca^2+^ levels
H_2_-DCFDA 10 µM	488	520	Intracellular ROS levels
JC1 5 µg/mL	488	520/590	Mitochondrial membrane potential

## Supplementary Materials

Supplementary materials can be found at https://www.mdpi.com/1422-0067/21/10/3638/s1.
