# Supplementary material for: Antioxidant Strategy to Prevent Simulated Microgravity-Induced Effects on Bone Osteoblasts
Source: Int J Mol Sci. 2020 May 21;21(10):3638. doi: 10.3390/ijms21103638 (PMC7279347; doi:10.3390/ijms21103638)
Supplement: Supplementary file 1 [file ijms-21-03638-s001.zip › ijms-785389-Supplementary material-Fig. 1.pdf]

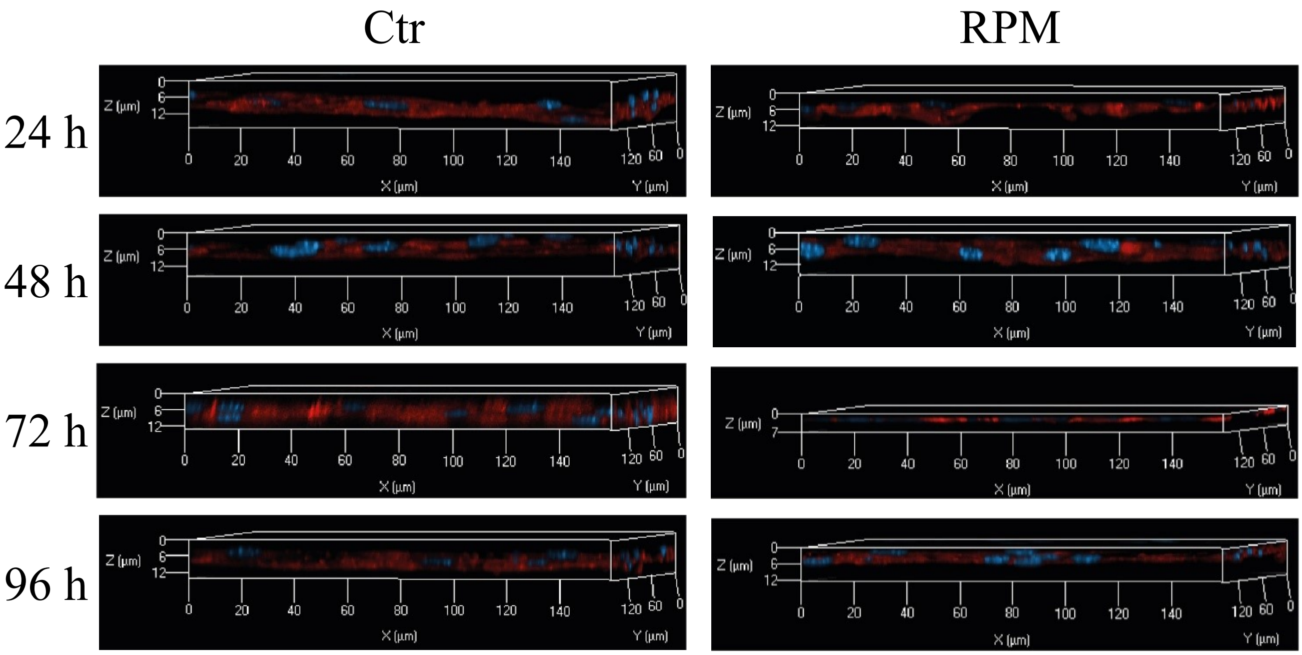

Supplementary Figure 1. Representative orthogonal (X-Y-Z) reconstruction of z-stack images from control cells at 1g (Ctr) or cells exposed to s-microgravity (RPM) at different times (24, 48, 72 and 96 h), stained with Alexa Fluor 546 Phalloidin (for f-actin) and DAPI (for nuclei).
