# Supplementary material for: Antioxidant Strategy to Prevent Simulated Microgravity-Induced Effects on Bone Osteoblasts
Source: Int J Mol Sci. 2020 May 21;21(10):3638. doi: 10.3390/ijms21103638 (PMC7279347; doi:10.3390/ijms21103638)
Supplement: Supplementary file 1 [file ijms-21-03638-s001.zip › ijms-785389-Supplementary material-Tables Rev2.pdf]

Supplementary Tables

Tables S1A and B. A. Intracellular ROS Levels. Data are expressed as f/cell number and are means ± SEM from three independent experiments (each containing eight repetitions). The data collected in these tables came from experiments in which 100 µM Trolox (Trolox) was used. Trolox was solubilized in dimethyl sulphoxide (DMSO) at concentration of 100 mM than diluted in cell medium at a final concentration of 100 µM. In each experimental set, samples were also treated with DMSO (1 µl dimethyl sulphoxide in 1 ml medium) to exclude its possible effect or toxicity on cells. **B**. Statistical significance calculated by Student's t-test (Prism5 software, GraphPad, San Diego, CA, USA).

A

Time	Ctr	Ctr + DMSO	Ctr + Trolox	RPM	RPM + DMSO	RPM + Trolox
**24 h**	0.173 ± 0.010	0.175 ± 0.003	0.161 ± 0.006	0.392 ± 0.012	0.381 ± 0.012	0.168 ± 0.020
**48 h**	0.190 ± 0.024	0.201 ± 0.019	0.197 ± 0.005	0.535 ± 0.025	0.569 ± 0.010	0.203 ± 0.006
**72 h**	0.179 ± 0.037	0.189 ± 0.021	0.181 ± 0.005	0.453 ± 0.042	0.413 ± 0.010	0.135 ± 0.035
**96 h**	0.192 ± 0.018	0.197 ± 0.001	0.166 ± 0.011	0.447 ± 0.034	0.458 ± 0.015	0.159 ± 0.036

B

Sample comparison	24 h	48 h	72 h	96 h
Ctr *vs* RPM	***p< 0.001	***p< 0.001	***p< 0.001	***p< 0.001
Ctr+DMSO *vs* RPM+DMSO	***p< 0.001	***p< 0.001	***p< 0.001	***p< 0.001
Ctr+Trolox *vs* RPM+Trolox	p = 0.754	p = 0.485	p = 0.263	p = 0.862
Ctr+DMSO *vs* Ctr+Trolox	p = 0.105	p = 0.849	p = 0.730	p = 0.050
RPM+DMSO *vs* RPM+Trolox	***p< 0.001	***p< 0.001	**p< 0.005	**p< 0.005

Tables S2A and B. A. Intracellular Ca^{2+} Levels. Data are expressed as f/cell number and are means ± SEM from three independent experiments (each containing eight repetitions). **B**. Statistical significance calculated by Student's t-test (Prism5 software, GraphPad, San Diego, CA, USA).

A

Time	Ctr	Ctr + DMSO	Ctr + Trolox	RPM	RPM + DMSO	RPM + Trolox
**24 h**	0.195 ± 0.007	0.181 ± 0.005	0.196 ± 0.020	0.274 ± 0.011	0.264 ± 0.012	0.220 ± 0.013
**48 h**	0.171 ± 0.005	0.167 ± 0.005	0.156 ± 0.004	0.273 ± 0.016	0.269 ± 0.010	0.159 ± 0.006
**72 h**	0.199 ± 0.004	0.195 ± 0.002	0.206 ± 0.008	0.218 ± 0.018	0.201 ± 0.010	0.207 ± 0.008
**96 h**	0.175 ± 0.036	0.183 ± 0.001	0.183 ± 0.009	0.215 ± 0.005	0.208 ± 0.015	0.211 ± 0.016

B

Sample comparison	24 h	48 h	72 h	96 h
Ctr *vs* RPM	***p< 0.001	***p< 0.001	p = 0.361	p = 0.333
Ctr+DMSO *vs* RPM+DMSO	**p< 0.005	***p< 0.001	p = 0.588	p = 0.172
Ctr+Trolox *vs* RPM+Trolox	p = 0.371	p = 0.699	p = 0.934	p = 0.202
Ctr+DMSO *vs* Ctr+Trolox	p = 0.507	p = 0.161	p = 0.253	p> 0.999
RPM+DMSO *vs* RPM+Trolox	*p< 0.05	***p< 0.001	p = 0.664	p = 0.898

Tables S3A and B. A. Mitochondrial membrane potential. Data are expressed as fred/fgreen ratio and are means ± SEM from three independent experiments (each containing eight repetitions). **B**. Statistical significance calculated by Student's t-test (Prism5 software, GraphPad, San Diego, CA, USA).

A

Time	Ctr	Ctr + DMSO	Ctr + Trolox	RPM	RPM + DMSO	RPM + Trolox
**24 h**	0.698 ± 0. 020	0.701 ± 0.003	0.690 ± 0.010	0.696 ± 0.030	0.699 ± 0.010	0.673 ± 0.110
**48 h**	0.718 ± 0.027	0.711 ± 0.023	0.588 ± 0.110	0.577 ± 0.030	0.566 ± 0.031	0.705 ± 0.047
**72 h**	0.573 ± 0.015	0.564 ± 0.015	0.501 ± 0.010	0.531 ± 0.010	0.519 ± 0.010	0.552 ± 0.028
**96 h**	0.608 ± 0.024	0.617 ± 0.02	0.627 ± 0.020	0.615 ± 0.023	0.635 ± 0.02	0.635 ± 0.020

B

Sample comparison	24 h	48 h	72 h	96 h
Ctr *vs* RPM	p = 0.958	**p< 0.005	*p< 0.05	p = 0.850
Ctr+DMSO *vs* RPM+DMSO	p = 0.857	**p< 0.005	*p< 0.05	p = 0.559
Ctr+Trolox *vs* RPM+Trolox	p = 0.885	p = 0.383	p = 0.161	p = 0.791
Ctr+DMSO *vs* Ctr+Trolox	p = 0.352	p = 0.335	*p< 0.05	p = 0.753
RPM+DMSO *vs* RPM+Trolox	p = 0.760	*p< 0.05	*p< 0.05	p> 0.999

Tables S4A and B. A. Glucose levels in the medium. Data are expressed as (μg glucose/ml)/cell number and are means ± SEM from three independent experiments (each containing five repetitions). **B**. Statistical significance calculated by Student's t-test (Prism5 software, GraphPad, San Diego, CA, USA).

A

**Time**	**Ctr**	**Ctr + DMSO**	**Ctr +Trolox**	**RPM**	**RPM + DMSO**	**RPM + Trolox**
**24 h**	0.231 ± 0.001	0.215 ± 0.008	0.219 ± 0.019	0.318 ± 0.005	0.323 ± 0.001	0.262 ± 0.048
**48 h**	0.144 ± 0.003	0.139 ±0.003	0.135 ± 0.003	0.247 ± 0.004	0.235 ± 0.005	0.148 ± 0.003
**72 h**	0.109 ± 0.001	0.103 ± 0.002	0.103 ± 0.001	0.175 ±0.003	0.166 ±0.001	0.108 ± 0.002
**96 h**	0.106 ± 0.001	0.101 ± 0.003	0.101 ± 0.001	0.171 ± 0.004	0.163 ± 0.003	0.104 ± 0.002

B

**Sample comparison**	**24 h**	**48 h**	**72 h**	**96 h**
Ctr *vs* RPM	***p< 0.001	***p< 0.001	***p< 0.001	***p< 0.001
Ctr+DMSO *vs* RPM+DMSO	*** p< 0.001	***p< 0.001	***p< 0.001	***p< 0.001
Ctr+Trolox *vs* RPM+Trolox	p = 0.452	* p< 0.05	p = 0.089	p = 0.251
Ctr+DMSO *vs* Ctr+Trolox	p = 0.856	p = 0.399	p> 0.9999	p> 0.9999
RPM+DMSO *vs* RPM+Trolox	***p < 0.001	***p< 0.001	***p< 0.001	***p< 0.001

Tables S5A and B. A. Lactate levels in the medium. Data are expressed as (nmol lactate/ml)/cell number and are means ± SEM from three independent experiments (each containing five repetitions). **B**. Statistical significance calculated by Student's t-test (Prism5 software, GraphPad, San Diego, CA, USA).

A

**Time**	**Ctr**	**Ctr + DMSO**	**Ctr + Trolox**	**RPM**	**RPM + DMSO**	**RPM + Trolox**
**24 h**	0.417 ± 0.002	0.401 ± 0.021	0.341 ± 0.020	0.629 ± 0.020	0.617 ± 0.023	0.284 ± 0.021
**48 h**	0.357 ± 0.005	0.323 ± 0.017	0.271 ± 0.017	0.571 ± 0.032	0.565 ± 0.009	0.249 ± 0.022
**72 h**	0.215 ± 0.009	0.209 ± 0.015	0.201 ± 0.015	0.445 ± 0.025	0.431 ± 0.030	0.200 ± 0.029
**96 h**	0.195 ± 0.003	0.185 ± 0.013	0.179 ± 0.019	0.384 ± 0.019	0.373 ± 0.017	0.148 ± 0.018

B

**Sample comparison**	**24 h**	**48 h**	**72 h**	**96 h**
Ctr *vs* RPM	***p< 0.001	***p< 0.001	***p< 0.001	***p< 0.001
Ctr+DMSO *vs* RPM+DMSO	***p< 0.001	***p< 0.001	***p< 0.001	***p< 0.001
Ctr+Trolox *vs* RPM+Trolox	p = 0.359	p = 0.473	p = 0.977	p = 0.302
Ctr+DMSO *vs* Ctr+Trolox	p = 0.107	p = 0.097	p = 0.725	p = 0.807
RPM+DMSO *vs* RPM+Trolox	***p< 0.001	***p< 0.001	***p< 0.001	***p< 0.001

Tables S6A and B. A. Cell Height. Data are expressed as μm and are means ± SEM from three independent experiments (each containing five repetitions). **B**. Statistical significance calculated by Student's t-test (Prism5 software, GraphPad, San Diego, CA, USA).

A

**Time**	**Ctr**	**Ctr + DMSO**	**Ctr + Trolox**	**RPM**	**RPM + DMSO**	**RPM + Trolox**
**24 h**	6.10 ± 0.11	6.05 ± 0.12	5.93 ± 0.20	6.32 ± 0.17	6.27 ± 0.21	5.77 ± 0.13
**48 h**	6.41 ± 0.09	6.41 ± 0.11	6.15 ± 0.11	6.88 ± 0.10	6.93 ± 0.09	6.46 ± 0.10
**72 h**	6.21 ± 0.12	6.12 ± 0.10	6.09 ± 0.13	4.63 ± 0.14	4.51 ± 0.16	6.54 ±0.10
**96 h**	6.71 ± 0.19	6.82 ± 0.17	6.51 ± 0.12	5.03 ± 0.11	4.97 ± 0.10	6.32 ± 0.11

B

**Sample comparison**	**24 h**	**48 h**	**72 h**	**96 h**
Ctr *vs* RPM	p = 0.338	*p< 0.05	**p< 0.01	**p< 0.01
Ctr+DMSO *vs* RPM+DMSO	p = 0.415	*p< 0.05	**p< 0.01	**p< 0.01
Ctr+Trolox *vs* RPM+Trolox	p = 0.235	p = 0.445	p = 0.504	p = 0.279
Ctr+DMSO *vs* Ctr+Trolox	p = 0.250	p = 0.233	p = 0.291	p = 0.296
RPM+DMSO *vs* RPM+Trolox	p = 0.137	*p< 0.05	**p< 0.01	**p< 0.01

Tables S7A and B. A. Actin filament mean length in a single cell. Data are expressed as arbitrary unit (a.u.) and are means ± SEM from three independent experiments (each containing five repetitions). **B**. Statistical significance calculated by Student's t-test (Prism5 software, GraphPad, San Diego, CA, USA).

A

**Time**	**Ctr**	**Ctr + DMSO**	**Ctr + Trolox**	**RPM**	**RPM+ DMSO**	**RPM + Trolox**

**24 h**	0.574 ± 0.06	0.560 ± 0.09	0.464 ± 0.05	0.577 ± 0.08	0.565 ± 0.09	0.435 ± 0.09
**48 h**	0.452 ± 0.05	0.460 ± 0.04	0.468 ± 0.06	0.771 ± 0.08	0.779 ± 0.09	0.621 ± 0.08
**72 h**	0.384 ± 0.01	0.391 ± 0.04	0.390 ± 0.04	0.673 ± 0.09	0.669 ± 0.04	0.372 ± 0.06
**96 h**	0.431 ±0.02	0.411 ±0.02	0.445 ± 0.07	0.661 ± 0.03	0.672 ± 0.03	0.379 ± 0.07

B

**Sample comparison**	**24 h**	**48 h**	**72 h**	**96 h**
Ctr *vs* RPM	p = 0.977	*p< 0.05	**p< 0.01	**p< 0.01
Ctr+DMSO *vs* RPM+DMSO	p = 0.382	*p< 0.05	**p< 0.01	**p< 0.01
Ctr+Trolox *vs* RPM+Trolox	p = 0.792	p = 0.202	p = 0.815	p = 0.542
Ctr+DMSO *vs* Ctr+Trolox	p = 0.404	p = 0.917	p = 0.987	p = 0.665
RPM+DMSO *vs* RPM+Trolox	p = 0.365	*p< 0.05	*p< 0.05	*p< 0.05

Tables S8A and B. A. Nucleus area. Data are expressed as μm^2 and are means ± SEM from three independent experiments (each containing five repetitions). **B**. Statistical significance calculated by Student's t-test (Prism5 software, GraphPad, San Diego, CA, USA).

A

**Time**	**Ctr**	**Ctr + DMSO**	**Ctr + Trolox**	**RPM**	**RPM + DMSO**	**RPM + Trolox**
**24 h**	141.1 ± 9.0	139.7 ± 7.2	142.2 ± 6.8	128.4 ± 13.1	136.3 ± 8.1	119.1 ± 9.1
**48 h**	137.1 ±2.6	135.7 ± 2.1	129.4 ± 2.6	150.4 ±3.8	148.1 ± 3.3	130.6 ± 2.5
**72 h**	139.5 ± 6.7	137.1 ± 2.5	135.0 ± 3.1	136.4 ± 3.1	139.3 ± 4.1	143.4 ± 6.6
**96 h**	141.1± 5.1	143.1 ± 2.9	149.0 ± 6.7	137.1± 3.3	140.5 ± 2.9	133.7 ± 6.2

B

**Sample comparison**	**24 h**	**48 h**	**72 h**	**96 h**
Ctr *vs* RPM	p = 0.469	*p< 0.05	p = 0.6961	p = 0.546
Ctr+DMSO *vs* RPM+DMSO	p = 0.769	*p< 0.05	p = 0.671	p = 0.561
Ctr+Trolox *vs* RPM+Trolox	p = 0.112	p = 0.756	p = 0.314	p = 0.169
Ctr+DMSO *vs* Ctr+Trolox	p = 0.813	p = 0.133	p = 0.626	p = 0.464
RPM+DMSO *vs* RPM+Trolox	p = 0.231	*p< 0.05	p = 0.626	p = 0.377

Tables S9A and B. A. Nucleus roundness. Data are expressed as R (see Materials and Methods and Fig. 1) and are means ± SEM from five independent experiments (each containing three repetitions). **B**. Statistical significance calculated by Student's t-test (Prism5 software, GraphPad, San Diego, CA, USA).

A

**Time**	**Ctr**	**Ctr+DMSO**	**Ctr+Trolox**	**RPM**	**RPM+DMSO**	**RPM+Trolox**
**24 h**	0.85 ± 0.01	0.83 ± 0.02	0.87 ± 0.06	0.81 ±0.03	0.84 ± 0.06	0.79 ± 0.03
**48 h**	0.82 ± 0.01	0.78 ± 0.02	0.79 ± 0.01	0.86 ±0.01	0.86± 0.01	0.85 ± 0.01
**72 h**	0.80 ± 0.01	0.79 ± 0.01	0.83 ± 0.01	0.85 ± 0.01	0.87 ± 0.02	0.87 ± 0.01
**96 h**	0.79 ± 0.01	0.81 ± 0.01	0.80 ± 0.01	0.83± 0.01	0.85 ± 0.01	0.85 ± 0.01

B

**Sample comparison**	**24 h**	**48 h**	**72 h**	**96 h**
Ctr *vs* RPM	p = 0.275	*p< 0.05	*p< 0.05	*p< 0.05
Ctr+DMSO *vs* RPM+DMSO	p = 0.882	*p< 0.05	*p< 0.05	*p< 0.05
Ctr+Trolox *vs* RPM+Trolox	p = 0.299	*p< 0.05	*p< 0.05	*p< 0.05
Ctr+DMSO *vs* Ctr+Trolox	p = 0.561	p = 0.678	p = 0.05	p = 0.519
RPM+DMSO *vs* RPM+Trolox	p = 0.498	p = 0.519	p> 0.999	p> 0.999

Tables S10A and B. A. Western blot analysis of samples at 24 h. Data are the relative expression calculated as ratio between the optical density (OD) × mm^2 of each band and OD × mm^2 of the corresponding GAPDH band, used as loading control. Data are means ± SEM from three independent experiments. **B**. Statistical significance calculated by Student's t-test (Prism5 software, GraphPad, San Diego, CA, USA).

A

	**Ctr**	**RPM**	**Ctr + Trolox**	**RPM + Trolox**
**β1 integrin/ GAPDH**	0.70 ±0.11	2.2 ± 0.40	0.59 ± 0.12	0.6 ± 0.06
**β actin/ GAPDH**	0.85 ± 0.1	1.6 ± 0.15	0.71 ± 0.11	0.95 ± 0.12

B

**Sample comparison (β1 integrin/GAPDH)**	**24 h**	**Sample comparison (β actin/GAPDH)**	**24 h**
Ctr *vs* RPM	*p< 0.05	Ctr *vs* RPM	*p< 0.05
Ctr+Trolox *vs* RPM+Trolox	p = 0.944	Ctr+Trolox *vs* RPM+Trolox	p = 0.214

Tables S11A, B, C, D, E and F. A. Cell Proliferation. Data are expressed as live-cell number and are means ± SEM from three independent experiments (each containing three repetitions). **B**. Statistical significance in live cell number calculated by Student's t-test (Prism5 software, GraphPad, San Diego, CA, USA). **C**. Dead cell number expressed as means ± SEM from three independent experiments (each containing three repetitions). **D**. Statistical significance in dead cell number calculated by Student's t-test (Prism5 software, GraphPad, San Diego, CA, USA). **E**. Dead cell percentage expressed as means ± SEM from three independent experiments (each containing three repetitions). **F**. Statistical significance in percentage of dead cell calculated by Student's t-test (Prism5 software, GraphPad, San Diego, CA, USA).

A

**Time**	**Ctr**	**Ctr + DMSO**	**Ctr + Trolox**	**RPM**	**RPM + DMSO**	**RPM + Trolox**
**24 h**	740000 ± 140000	728000 ± 117000	725000 ± 25000	627000 ± 12800	653000 ± 91000	775000 ± 33000
**48 h**	1262000 ± 87500	1211000 ± 65000	1150000 ± 100000	925000 ± 75000	917000 ± 32000	1163000 ± 112000
**72 h**	1491400 ± 120000	1513000 ± 97000	1612500 ± 62500	1095000 ± 95000	990000 ± 105000	1522000 ± 27500
**96 h**	1530000 ± 93000	1589000 ± 99100	1597000 ± 75000	1150000 ± 85000	1203000 ± 117000	1562000 ± 112500

B

**Sample comparison**	**24 h**	**48 h**	**72 h**	**96 h**
Ctr *vs* RPM	p = 0.583	*p< 0.05	*p< 0.05	*p< 0.05
Ctr+DMSO *vs* RPM+DMSO	p = 0.640	*p< 0.05	*p< 0.05	*p< 0.05
Ctr+Trolox *vs* RPM+Trolox	p = 0.294	p = 0.935	p = 0.256	p = 0.809
Ctr+DMSO *vs* Ctr+Trolox	p = 0.981	p = 0.636	p = 0.437	p = 0.952
RPM+DMSO *vs* RPM+Trolox	p = 0.276	*p< 0.05	*p< 0.05	*p< 0.05

C

**Time**	**Ctr**	**Ctr + DMSO**	**Ctr + Trolox**	**RPM**	**RPM + DMSO**	**RPM + Trolox**
**24 h**	22200 ± 1110	21840 ± 1092	21750 ± 1050	19810 ± 2100	20896 ± 1940	24800 ±1550
**48 h**	44170 ± 2325	42333 ± 2900	41959 ± 3150	29600 ± 2850	29934 ± 1467	39316 ± 2900
**72 h**	59656 ± 2889	63546 ± 3890	64500 ± 2955	43800 ± 2500	41580 ± 2900	60500 ± 3157
**96 h**	61200 ± 2448	65540 ± 3570	63888 ± 2194	46000 ± 2350	48120 ± 2110	62580 ± 3126

D

**Sample comparison**	**24 h**	**48 h**	**72 h**	**96 h**
Ctr *vs* RPM	p = 0.371	*p< 0.05	*p< 0.05	*p< 0.05
Ctr+DMSO *vs* RPM+DMSO	p = 0.693	*p< 0.05	*p< 0.05	*p< 0.05
Ctr+Trolox *vs* RPM+Trolox	p = 0.179	p = 0.571	p = 0.407	p = 0.749
Ctr+DMSO *vs* Ctr+Trolox	p = 0.955	p = 0.935	p = 0.855	p = 0.714
RPM+DMSO *vs* RPM+Trolox	p = 0.191	*p< 0.05	*p< 0.05	*p< 0.05

E

**Time**	**Ctr**	**Ctr + DMSO**	**Ctr + Trolox**	**RPM**	**RPM + DMSO**	**RPM + Trolox**
**24 h**	2.91 ± 0.14	2.91 ± 0.15	2.91 ± 0.14	3.06 ± 0.32	3.10 ± 0.28	3.10 ± 0.19
**48 h**	3.38 ± 0.18	3.38 ± 0.23	3.52 ± 0.26	3.10 ± 0.29	3.16 ± 0.15	3.27 ± 0.24
**72 h**	3.74 ± 0.18	4.01 ± 0.25	3.84 ± 0.18	3.85 ± 0.22	4.03 ± 0.28	3.82 ± 0.19
**96 h**	3.84 ± 0.15	3.96 ± 0.22	3.85 ± 0.13	3.85 ± 0.19	3.84 ± 0.17	3.85 ± 0.19

F

**Sample comparison**	**24 h**	**48 h**	**72 h**	**96 h**
Ctr *vs* RPM	p = 0.689	p = 0.458	p = 0.7185	p = 0.969
Ctr+DMSO *vs* RPM+DMSO	p = 0.582	p = 0.468	p = 0.960	p = 0.688
Ctr+Trolox *vs* RPM+Trolox	p = 0.466	p = 0.519	p = 0.943	p> 0.999
Ctr+DMSO *vs* Ctr+Trolox	p> 0.999	p = 0.707	p = 0.610	p = 0.689
RPM+DMSO *vs* RPM+Trolox	p> 0.999	p = 0.717	p = 0.569	p = 0.971
